# Severe community-acquired pneumonia: an integral approach

**DOI:** 10.1186/s40560-026-00854-x

**Published:** 2026-01-31

**Authors:** Arsah Asis, Darren McMahon, Shigeki Fujitani, Pedro Povoa, Alejandro Rodriguez, Luis Felipe Reyes, Ignacio Martin-Loeches

**Affiliations:** 1 Department of Intensive Care Medicine, Multidisciplinary Intensive Care Research Organization (MICRO), St James’ Hospital, Dublin, Ireland; 2 School of Medicine, Trinity College Dublin, Dublin, Ireland; 3 Trinity Centre for Biomedical Engineering (TCBE), Dublin, Ireland; 4 Department of Emergency and Critical Care Medicine, St. Marianna University School of Medicine, Kanagawa, Japan; 5 NOVA Medical School, CHRC, NOVA University of Lisbon, Lisbon, Portugal; 6 Research Unit of Clinical Epidemiology, Department of Clinical Research, OUH Odense University Hospital, Odense, Denmark; 7 Department of Intensive Care, Hospital de São Francisco Xavier, ULSLO, Lisbon, Portugal; 8 Critical Care Unit, Joan XXIII University Hospital, Tarragona, Spain; 9 Clínica Universidad de La Sabana, Chía, Colombia; 10 Unisabana Center for Translational Science, School of Medicine, Universidad de La Sabana, Chía, Colombia; 11 ISARIC, Pandemic Sciences Institute, University of Oxford, Oxford, UK; 12 Universidad de La Sabana, Chía, Colombia

**Keywords:** Severe community-acquired pneumonia, Biomarkers, Diagnosis, Multiplex PCR, CURB-65, Antimicrobial stewardship, Corticosteroids, Immunomodulation, Intensive care

## Abstract

**Background:**

Severe community-acquired pneumonia represents the most critical manifestation of pneumonia acquired outside the hospital and remains a major cause of intensive care admission and death worldwide. It is frequently complicated by acute respiratory failure, circulatory shock, and multiple organ dysfunction, with mortality commonly exceeding 25–40%. Despite progress in vaccination programmes, microbiological diagnostics, and critical care support, major uncertainties persist regarding early recognition, pathogen identification, antimicrobial selection and duration, management of co-infections, and assessment of treatment response. These challenges are amplified by increasing host heterogeneity and antimicrobial resistance.

**Main body:**

Contemporary epidemiological studies consistently demonstrate that mortality in severe community–acquired pneumonia is closely linked to the severity of organ dysfunction rather than to pulmonary involvement alone. In response, the recent international guideline definitions incorporate both respiratory and systemic criteria to standardise identification and guide early triage. However, these frameworks also reveal substantial variability related to host factors that influence clinical presentation, microbiological findings, and outcomes. Advanced age, sex, chronic cardiopulmonary disease, diabetes, frailty, and prior healthcare exposure modify risk, while immunocompromised populations—including transplant recipients, patients with malignancy, individuals receiving immunomodulatory therapies, and people living with Human Immunodeficiency Virus in resource-limited settings—face distinct diagnostic and therapeutic challenges.

Accurate microbiological diagnosis remains difficult, with mixed or unidentified infections frequently encountered. Structured respiratory sampling and molecular diagnostic techniques, particularly lower respiratory tract multiplex polymerase chain reaction panels, have improved pathogen detection and support earlier optimisation of antimicrobial therapy. Biomarkers such as procalcitonin and C-reactive protein provide complementary information on disease trajectory and response to treatment, enabling more confident reassessment and earlier discontinuation of antibiotics when aligned with clinical improvement. Severity stratification using combined clinical scores, radiological extent, and laboratory markers refines prognostication and informs treatment intensity. Empiric antimicrobial therapy should be initiated promptly and individualised according to patient-specific risk factors for resistant pathogens, with systematic reassessment within 24–48 h. Adjunctive strategies, including advanced respiratory and haemodynamic support, corticosteroids in selected inflammatory phenotypes, and emerging host-directed therapies such as intravenous immunoglobulins, form part of a personalised management approach.

**Conclusions:**

An integrated, multidisciplinary strategy that links early recognition, diagnostic precision, severity assessment, and personalised therapy offers a coherent pathway to improve outcomes and promote responsible antimicrobial use in severe community-acquired pneumonia.

## Background

Community-acquired pneumonia (CAP) remains one of the leading causes of morbidity and mortality worldwide, in low income countries, and continues to pose a massive burden on healthcare systems. Lower respiratory tract infections caused by CAP accounted for nearly 2.38 million deaths globally in 2016 [1]. Among CAP cases, a substantial fraction progress to its most dangerous form, severe community-acquired pneumonia (sCAP) which frequently leads to acute respiratory failure, septic shock, and multi-organ dysfunction. In high income countries, about 2–20% of CAP patients require ICU admission due to severity. Once admitted to the ICU with sCAP, mortality remains alarmingly high. Cohort studies report case-fatality rates ranging from 21% up to 54%, with many analyses clustering around 25–35% [1, 2]. These sobering figures underscore that despite advances in diagnostics, vaccination, antimicrobial therapy and critical care supports, sCAP remains a life-threatening condition, a stark reminder of the urgent need for better risk stratification, rapid and precise microbiological diagnostics, personalised therapeutic frameworks, and improved preventive measures.

Recognition and management are further complicated by diagnostic and therapeutic uncertainties, including undetermined aetiology at presentation, marked host-related heterogeneity, and ambiguity around optimal antimicrobial duration. Substantial variation in clinical, host, pathogen(s), and treatment factors shapes disease presentation and response. Age, sex, and comorbidities such as chronic cardiopulmonary disease, diabetes, and frailty modify risk profiles, while immunosuppressed groups including solid-organ transplant recipients, patients with cancer or on immunomodulatory therapy, and individuals living with HIV in low- and middle-income countries exhibit distinct trajectories and additional diagnostic challenges [3–5]. Clinicians across healthcare settings therefore face recurring questions during the course of illness such as, whether the patient is at risk of progressing to sCAP, whether infection is truly present and by which pathogen, whether infection has resolved with treatment, and whether refractory infection or infectious/non-infectious complications have emerged. These reflect the dynamic and often challenging decision-making inherent to sCAP [4]. The lack of definitive diagnostic markers, variability in biomarker interpretation, and challenges in defining antibiotic duration further compound these issues. Biomarker-embedded algorithms can help but must always be applied within clinical context and importantly never as stand-alone toll [6, 7]. Such uncertainty reinforces the need for a structured, integral approach that links early recognition, precise microbiologic diagnosis with biomarker support, severity stratification, and individualised therapy guided by contemporary international guidelines and systematic reassessment at 24–48 h [3, 4, 8, 9]. sCAP should not be regarded as a localised pulmonary infection but as a systemic inflammatory disease with multi-organ involvement, a final common pathway of dysregulated host–pathogen interaction leading to sepsis, Acute Respiratory Distress Syndrome (ARDS), and multi-organ failure [8].

Adding to this complexity is the fact that community-acquired pneumonia is understood differently across clinical specialties, shaped by their distinct conceptual frameworks and priorities. CAP remains a heterogeneous clinical syndrome whose definition, assessment, and management vary according to care setting and disease severity. Although international guidelines offer standardised diagnostic and therapeutic criteria, substantial variation persists in how clinicians interpret and operationalise them in practice. For respiratory physicians, attention is often directed toward the extent of parenchymal involvement and the physiological impact on gas exchange whereas infectious diseases specialists tend to prioritise microbial aetiology, antimicrobial selection, and resistance profiles. Intensivists, in turn, often conceptualise severe CAP within the broader framework of sepsis, shock, and evolving multi-organ dysfunction. These divergent perspectives drive heterogeneity in diagnostic pathways and therapeutic choices, underscoring the necessity for harmonised, multidisciplinary strategies to optimise patient outcomes [5, 10].

Given these conceptual and operational inconsistencies, there is a critical need for a unified framework that harmonises infection recognition, diagnosis, and management across clinical specialties. This review uses these uncertainties as a platform to examine and synthesise current evidence regarding the definition and epidemiology of sCAP, the diagnostic approach including the emerging role of biomarkers, the utility of risk-stratification tools for assessing disease severity, and the spectrum of management strategies encompassing antimicrobial therapy, co-treatments, and adjunctive interventions. By integrating findings from the recent multicentre studies and contemporary clinical guidelines, this review aims to promote a cohesive and multidisciplinary strategy for the recognition and treatment of sCAP, bridging the perspectives of respiratory medicine, infectious diseases, and critical care to improve outcomes in this high-risk population.

### Epidemiology

Classical population studies established the epidemiologic foundations of CAP and demonstrated wide variation in outcomes depending on care setting. In a landmark study, Fine et al. developed the Pneumonia Severity Index (PSI), showing that overall mortality for CAP ranges from < 1% among low-risk outpatients to approximately 27% in the highest-risk group requiring hospitalisation [11]. Subsequent work confirmed that mortality escalates sharply with severity, typically < 5% in ward-treated patients, 10–20% among those needing high-dependency care, and up to 30–50% in cases admitted to ICU [9, 12, 13]. These early multicentre datasets remain highly relevant illustrating that CAP severity and outcome correlate with age, comorbidities, and physiologic instability at presentation.

Within hospital cohorts, sCAP represents approximately 10–20% of all CAP admissions and accounts for most pneumonia-related ICU deaths. In a large multicentre analysis involving 664 patients admitted to ICU with sCAP, Ferrer et al. found that 30–day mortality was 16% in those without shock or invasive mechanical ventilation (IMV), 25% in those with shock alone, 30% with IMV alone, and 38% when both were present. Median ICU stay extended from 3 days in non-shocked patients to 11 days in those with combined shock and IMV, highlighting the additive risk of cardiorespiratory failure [14].

The data from the Global Burden of Disease (GBD) 2019 project reinforce that lower respiratory infections including CAP remain a leading cause of death in all age groups, responsible for 2.6 million deaths worldwide in 2019 despite improved vaccination and access to antimicrobials [15]. Similarly, Jain et al. demonstrated in a large prospective US cohort that pneumonia requiring hospitalisation continues to be associated with significant morbidity, particularly in elderly patients and those with chronic conditions [16]. However, because the data from many low- and middle-income countries remain limited, the true mortality burden is likely underestimated.

### Definition

The definition of severe community-acquired pneumonia (sCAP) has evolved from broad, radiology-driven descriptions to consensus-based frameworks integrating respiratory failure and systemic organ dysfunction, with the aim of standardising patient identification, optimising ICU triage, and harmonising clinical research inclusion criteria.

A major milestone was the 2007 American Thoracic Society/Infectious Diseases Society of America (ATS/IDSA) guideline, which introduced a unified severity classification distinguishing severe from non-severe CAP using major and minor criteria (Table 1) [17]. Major criteria included the need for invasive mechanical ventilation and septic shock requiring vasopressors, while minor criteria encompassed physiological and radiological markers such as multilobar infiltrates, hypotension, and tachypnoea. These criteria were refined in the 2019 ATS/IDSA update, defining sCAP as the presence of one major or three or more minor criteria, thereby shifting emphasis away from radiographic extent alone toward markers of respiratory failure and systemic dysfunction [4], Validation studies have consistently shown that these criteria reliably predict ICU admission and mortality, supporting their clinical utility and practicality for bedside decision-making [18].

**Table 1:** 2019 ATS/IDSA criteria for severe community-acquired pneumonia [4]

Category	Criteria	Clinical description/comment
Major criteria	Septic shock requiring vasopressors	Indicates circulatory failure secondary to infection
	Need for invasive mechanical ventilation	Indicates severe respiratory failure requiring intubation and ventilatory support
Minor criteria	Respiratory rate ≥ 30 breaths/min	Reflects respiratory distress and compensatory tachypnoea
	PaO₂/FiO₂ ≤ 250	Reflects impaired gas exchange and hypoxemia
	Multilobar infiltrates	Radiologic indicator of disease extent and severity
	Confusion/disorientation	Marker of systemic inflammatory response and cerebral hypoperfusion
	Uraemia (BUN ≥ 20 mg/dL)	Reflects renal dysfunction or dehydration
	Leukopenia (WBC < 4 × 10⁹/L)	Suggests severe infection or sepsis-induced marrow suppression
	Thrombocytopenia (platelet count < 100 × 10⁹/L)	Marker of systemic inflammatory response or DIC
	Hypothermia (core temperature < 36 °C)	Indicator of severe systemic infection and poor host response
	Hypotension requiring aggressive fluid resuscitation	Reflects circulatory compromise prior to shock

*BUN* Blood urea nitrogen*, DIC* Disseminated intravascular coagulation*, PaO₂/FiO₂* Ratio of arterial oxygen partial pressure to fraction of inspired oxygen*, WBC* White blood cell count

Building on this evolution, the joint European Respiratory Society (ERS), European Society of Intensive Care Medicine (ESICM), European Society of Clinical Microbiology and Infectious Diseases (ESCMID), and Latin American Thoracic Association (ALAT) international guidelines further reframed sCAP as a syndrome of critical infection, defined by the coexistence of pulmonary failure and systemic organ dysfunction rather than isolated respiratory compromise [5]. This conceptualisation aligns sCAP more closely with sepsis and acute respiratory distress syndrome paradigms and recognises its heterogeneous bacterial, viral, and mixed aetiologies. The framework incorporates microbiological confirmation where feasible, host-response profiling, biomarker integration, and structured organ-dysfunction assessment to support early ICU triage and therapeutic precision (Table 2) [5].

**Table 2:** ERS/ESICM/ESCMID/ALAT framework for severe community-acquired pneumonia (sCAP) [5]

Domain	Key principle and rationale
Definition of severity	Severe CAP is characterised by acute respiratory failure requiring advanced oxygenation or ventilatory support (HFNO, NIV, or IMV), or circulatory failure requiring vasopressors. These features reflect the transition from isolated lung infection to systemic critical illness, helping clinicians identify patients needing urgent escalation and ICU admission
Diagnostic strategy	Early acquisition of microbiological specimens (including sputum or tracheal aspirates, urine antigens, blood cultures, and multiplex PCR panels) before starting antimicrobials increases diagnostic yield and supports targeted therapy. Results should be reassessed within 24–48 h to refine treatment rather than continuing broad-spectrum therapy empirically
Role of biomakers	Biomarkers such as PCT can complement clinical judgment by assisting in differentiating bacterial from viral infection, guiding antimicrobial duration, and monitoring response. However, they should be interpreted within a broader clinical context and not act as stand-alone decision-makers
Empirical antimicrobial therapy	Initial broad-spectrum antimicrobial therapy covering both typical and atypical pathogens (e.g., β-lactam plus macrolide) is recommended to avoid delays in appropriate treatment, which are strongly associated with mortality. Local resistance epidemiology should guide drug choice
Viral and Mixed Infections Management	Because viral and mixed aetiologies are increasingly recognised in sCAP, routine viral testing (influenza, SARS-CoV-2, RSV) is encouraged. Early initiation of antivirals when indicated may reduce progression and complications
Host and organ dysfunction assessment	Severity assessment should encompass markers of systemic involvement (e.g., shock, ARDS, acute kidney injury, neurological impairment), recognising that sCAP is a multisystem disorder rather than solely a pulmonary process
Adjunctive co-therapies	Corticosteroids can be considered in patients with sCAP complicated by shock, with evidence supporting hydrocortisone use in carefully selected populations. Their role is to improve haemodynamic stability
Duration and de-escalation of antimicrobials	Shorter treatment courses (5–7 days) are generally adequate for patients who demonstrate clinical improvement, reducing toxicity and antimicrobial resistance. Biomarker-guided strategies and culture results can support timely narrowing or discontinuation
Multidisciplinary care	Optimal sCAP management benefits from close collaboration among respiratory physicians, infectious-diseases specialists, and intensivists to align diagnostic, antimicrobial, and organ-support decisions and ensure rapid escalation when required

*ARDS* Acute Respiratory Distress Syndrome, *CAP* Community-Acquired Pneumonia, *CRP* C-reactive Protein, *HFNO* HighFlow Nasal Oxygen, *ICU* Intensive Care Unit, *IMV* Invasive Mechanical Ventilation, *NIV* Non-Invasive Ventilation, *PCR* Polymerase Chain Reaction, *PCT* Procalcitonin, *RSV* Respiratory Syncytial Virus, *sCAP* Severe Community-Acquired Pneumonia

Within this context, severity stratification remains essential for prognostication and escalation of care. Biomarkers, such as procalcitonin (PCT) provide dynamic insight into host response, with persistently elevated or non-declining levels associated with treatment failure and mortality in CAP and sCAP [6, 7, 19]. However, biomarker interpretation must be integrated with physiological assessment and clinical scoring systems to accurately reflect disease severity.

Among clinical tools, CURB-65 remains widely used and captures systemic involvement beyond the lung through renal, circulatory, and neurological parameters [8]. In sCAP, CURB-65 is particularly informative when combined with biomarker trajectories, as discordance between clinical scores and inflammatory markers may signal occult organ dysfunction or recovery [6, 7, 19].

Additional CAP-specific tools, including the sCAP score and SMART-COP, further refine risk stratification and have been externally validated for predicting ICU admission, need for advanced respiratory or vasopressor support, and mortality [36, 37]. From a critical care perspective, ICU-oriented frameworks such as Sepsis-3**,** with SOFA-based organ dysfunction assessment, align conceptually with sCAP as a critical infection syndrome [20, 21]. From a critical care perspective, ICU-oriented frameworks such as Sepsis-3*,* with SOFA-based organ dysfunction assessment, align conceptually with sCAP as a critical infection syndrome [22]. Narrative and systematic reviews support combining clinical scores with biomarkers to enhance decision-making across the care pathway [23].

Radiologic extent of disease also contributes independently to severity assessment. Bilateral or multilobar infiltrates are consistently associated with higher risks of sepsis, respiratory failure, and mortality. In a multicentre prospective study, Liapikou et al*.* demonstrated that bilateral involvement confers a worse prognosis than unilateral multilobar or localised disease, reinforcing the systemic nature of severe pneumonia [24].

Overall, these data support an integrated, multidimensional approach to defining and stratifying sCAP, combining clinical scores, radiologic findings, and biomarker kinetics. This framework, endorsed by the ERS/ESICM/ESCMID/ALAT guidelines, enables more precise risk stratification, optimised ICU triage, and individualised management pathways, moving beyond one-size-fits-all definitions toward precision assessment based on infection biology and host response [5].

### Diagnosis and stratification of severity

The diagnosis of sCAP remains one of the most challenging clinical tasks, primarily due to its multifactorial and polymicrobial aetiology but also several potential mimickers such as pulmonary oedema, lung cancer, pneumonitis from any reasons. A large multicentre cohort study by Ferrer et al. provided a comprehensive aetiologic analysis of sCAP whereby, among 664 ICU patients with severe pneumonia, mixed infections were responsible for a significant proportion. This included bacterial and viral combinations, and cases with two or more bacterial pathogens. Classic bacterial pathogens such as *Streptococcus pneumoniae*, *Staphylococcus aureus*, and *Haemophilus influenzae* remained prominent, but up to one-third of cases with documented aetiology involved viral or polymicrobial origins. Importantly, a causative agent was not identified in approximately 45–50% of cases despite extensive microbiological testing, reflecting ongoing diagnostic limitations [14]. Furthermore, Garces et al. demonstrated that failure to identify a causative pathogen in sCAP occurs more frequently in immunosuppressed patients where early targeted therapy to a defined pathogen could be of critical value given the high mortality in this cohort [25]. This high proportion of undiagnosed or mixed aetiologies underscores the central challenge in sCAP, in distinguishing the primary pathogen from colonisation or secondary infection especially in patients already receiving empirical antimicrobials. It also reinforces the need for rapid molecular diagnostics and repeated sampling in non-responders.

The growing burden of viral and co-infections further complicates diagnosis. Martin-Loeches et al. demonstrated an increasing incidence of bacterial co-infection from 11% in 2009 to 23% in 2015 among critically ill patients with influenza, which correlated strongly with adverse outcomes. Co-pathogens such as *S. aureus*, *S. pneumoniae*, and *Pseudomonas aeruginosa* frequently accompanied influenza and other viral pneumonias, amplifying systemic inflammation and respiratory failure [26]. These findings underscore the idea that a positive viral test does not exclude bacterial involvement and that co-infection must always be considered and treated promptly. Complementary European data from almost 20 years across 46 studies from Welte et al. further illustrate this microbial diversity while *S. pneumoniae* remains the leading pathogen in CAP, the spectrum includes atypicals (*Mycoplasma pneumoniae*, *Chlamydophila pneumoniae*), Gram-negative bacilli, and respiratory viruses, particularly in older or immunocompromised populations [27]. Together, these studies confirm that sCAP is rarely monomicrobial and that empirical therapy and diagnostics must account for this complexity.

The diagnostic complexity is further compounded by the under-recognition of viral pneumonia, partly due to the taxonomic diversity of respiratory viruses, which vary by genomic type (RNA or DNA), polarity (positive- or negative-sense), and the presence or absence of an envelope [24, 25]. Nevertheless, viral pneumonias regardless of their structural classification typically present with overlapping clinical and radiographic features such as fever, hypoxemia, and pulmonary infiltrates, rendering them largely indistinguishable from bacterial pneumonia [28]. Likewise, management decisions are virus-specific, determined by the pathogen identified (e.g., oseltamivir for influenza) and the patient's immune and physiologic status, not by viral structures. Importantly, this also includes avoiding corticosteroids in confirmed influenza, in line with current guideline recommendations [4, 29, 30]. Moreover, the severity of disease is driven more by the host inflammatory response and co-infection than by intrinsic viral characteristics such as envelope or genome type [30].

In response to these diagnostic challenges, the ERS/ESICM/ESCMID/ALAT guidelines recommend a structured, evidence-based diagnostic strategy. Specifically, clinicians should obtain lower respiratory tract samples such as sputum, endotracheal aspirates, or bronchoalveolar lavage for multiplex polymerase chain reaction (PCR) testing when clinically indicated. This recommendation reflects the superior diagnostic yield of lower respiratory specimens compared with nasopharyngeal or upper-airway swabs, particularly in critically ill or mechanically ventilated patients. Multiplex PCR enables rapid, simultaneous detection of common bacterial and viral pathogens, including mixed or atypical infections, and supports early refinement of empirical therapy [5]. Importantly, these assays should be performed prior to or shortly after antibiotic initiation to optimise diagnostic accuracy and to facilitate targeted antimicrobial management. Nevertheless, a recent meta-analysis found that while syndromic PCR substantially improves pathogen detection and accelerates antimicrobial optimisation, it does not translate into reductions in major clinical outcomes such as in-hospital mortality [31]. Integrating lower respiratory multiplex PCR into routine diagnostic pathways therefore represents a key step toward overcoming the persistent gap between infection recognition and microbiologic confirmation in severe community-acquired pneumonia [32, 33].

Although rapid lower-respiratory multiplex PCR panels improve pathogen detection and time to actionable microbiologic results, robust evidence for improved hard clinical outcomes in sCAP remains limited. The INHALE WP3 multicentre pragmatic RCT in hospital-acquired and ventilator-associated pneumonia showed that ICU-based syndromic PCR significantly improved early antibiotic stewardship compared with standard care, but failed to demonstrate non-inferiority for pneumonia cure at 14 days [34]. These findings suggest that while rapid molecular diagnostics are powerful tools for optimising antimicrobial use, their impact on mortality and clinical cure in sCAP still requires confirmation in dedicated trials.

### Biomarkers

Despite major advances in molecular diagnostics, a substantial proportion of patients with sCAP continue to have no identifiable pathogen, leaving clinicians to make therapeutic decisions amid uncertainty [14]. In this context, host-response biomarkers have emerged as valuable adjuncts to support diagnosis, prognostication, and antimicrobial stewardship [19, 35]. These findings are reinforced by more recent evidence, including a 2023 randomised trial by Papp et al. showing safe reductions in antibiotic duration, and a systematic review and meta-analysis by Dias et al. comparing PCT- and CRP-guided strategies, both confirming that biomarker-guided approaches can decrease antibiotic use without adversely affecting clinical outcomes [36, 37]. However, initial biomarker levels must be interpreted cautiously as PCT and CRP concentrations at presentation vary significantly with the duration of symptoms prior to admission, as demonstrated by Mendez et al. underscoring the need to apply biomarker algorithms within appropriate clinical context [38].

An ideal biomarker for pneumonia has been described as “SMART” which stands for Sensitive, Measurable, Actionable, Relevant, and Timely, reflecting its capacity to provide clinically actionable information within the constraints of real-world practice [19]. A comprehensive review by Póvoa and colleagues, summarising evidence from major randomised and observational studies, concluded that biomarker-guided antibiotic algorithms particularly those based on PCT and CRP can safely reduce antibiotic exposure and treatment duration without compromising clinical outcomes. Across these trials, the average reduction in antibiotic duration ranged from 2 to 4 days, with no significant difference in clinical cure or recurrence rates between biomarker-guided and standard-care groups [35]. However, a recent systematic review and meta-analysis comparing PCT and CRP-guided strategies reported a modestly higher rate of infection recurrence in biomarker-guided arms, highlighting the need for cautious patient selection and close clinical monitoring [37]. Importantly, the review emphasised that the benefit arises not from biomarker measurement alone, but from structured interpretation within clinical algorithms. Interestingly a recent study Overreliance on biomarkers without clinical context risks misinterpretation, *“a fool with a tool still remains a fool” *[6]. This principle is reinforced by the ADAPT-Sepsis Trial [39], a large randomised controlled study comparing procalcitonin- and CRP-guided antibiotic strategies, which demonstrated that biomarker guidance can safely reduce antibiotic exposure only when embedded within protocolised decision frameworks, without replacing clinical judgment. Collectively, these findings underscore that biomarkers should be used to augment, rather than substitute, clinical assessment, ideally within algorithms that combine biomarker-guided stopping rules with minimum or fixed-duration safeguards.

Beyond antimicrobial guidance, biomarkers also provide valuable prognostic insight. In a prospective case–control study evaluating predictors of treatment failure in CAP, Martin-Loeches and colleagues demonstrated that elevated inflammatory markers and more importantly, persistent biomarker elevation during therapy were independently associated with poor outcomes and ICU mortality [7]. Persistent PCT or CRP elevation after 72 h of therapy often indicates ongoing infection or treatment failure, prompting clinicians to reassess antimicrobial adequacy and investigate for complications such as empyema or secondary ventilator-associated pneumonia. Thus, biomarkers serve not only as therapeutic monitors but also as prognostic indicators in the management of sCAP. Importantly, relative changes in biomarker trajectories such as percentage decline in PCT or CRP are often more informative than absolute values, as they better reflect dynamic response to therapy and correlate with clinical improvement. This concept is supported by the Procalcitonin And Survival Study (PASS) group randomised controlled trial, in which PCT kinetics were evaluated as part of a biomarker-guided strategy for severe infections [40]. Reflecting these findings, the ERS/ESICM/ESCMID/ALAT guidelines recommend incorporating biomarkers such as PCT as supportive tools to help to guide antibiotic initiation and duration, and to evaluate treatment response while emphasising that biomarkers should complement, not replace clinical judgment and microbiological testing [5].

### Treatment strategies

The use of biomarkers such as PCT and CRP can refine diagnostic confidence and guide therapy duration, but they should never replace the need for prompt empiric treatment. The initial antimicrobial decision must therefore balance urgency, appropriateness, adequacy, and stewardship, guided by validated severity scores, biomarker kinetics, and epidemiologic risk. In their review of the challenges surrounding sCAP, Torres et al. emphasised that early appropriate antibiotic coverage remains the single most important modifiable factor influencing mortality. However, they also cautioned that the growing diversity of pathogens, increasing prevalence of multidrug-resistant (MDR) organisms, and variable host response complicate empiric selection [3].

These complexities highlight the need for supportive tools that can refine diagnostic certainty and guide therapeutic decisions. In this context, biomarkers such as PCT and CRP have emerged as valuable adjuncts, providing clinicians with additional information to help distinguish infectious from non-infectious processes, assess treatment response, and optimise antibiotic stewardship. Consequently, both the ATS/IDSA 2025 and ERS/ESICM/ESCMID/ALAT guidelines advocate a structured, approach that agree on several key principles [5, 41].

Key principles:

- Early, effective empiric therapy covering *Streptococcus pneumoniae* and atypical pathogens.
- Risk-factor adaptation by extending coverage to MRSA or *Pseudomonas aeruginosa* only when epidemiologic or clinical risk is clear.
- Pair diagnostics with therapy by sending lower-respiratory samples for culture and multiplex PCR before or immediately after the first dose.
- Systematic reassessment using host biomarkers and clinical response to shorten duration and narrow spectrum as soon as possible.

**Fig. 1:**
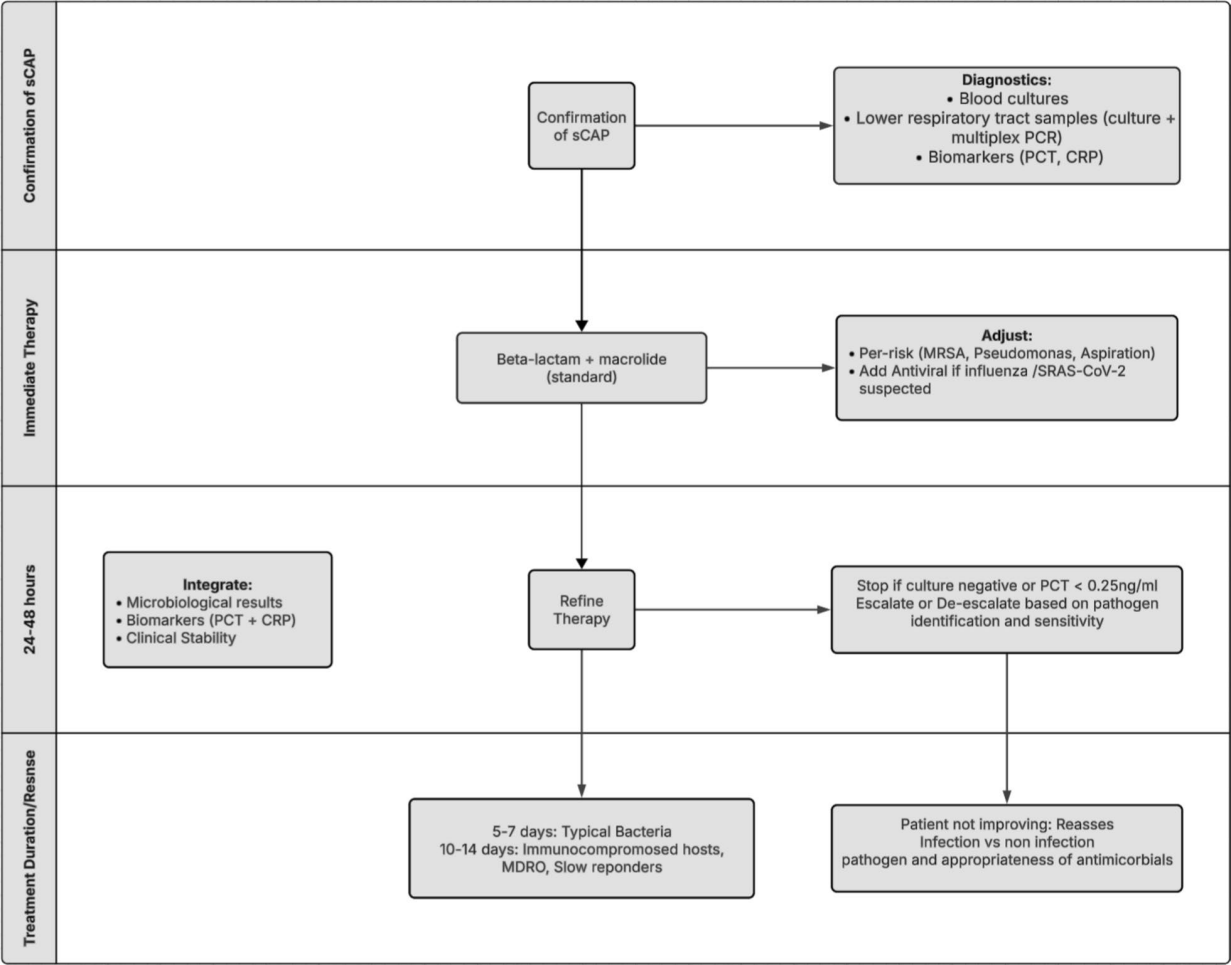
Integrated approach to empiric therapy in severe community–acquired pneumonia (sCAP) [10, 11]. Initial assessment confirms CAP or sCAP and includes baseline laboratory investigations. Empiric antimicrobial therapy is initiated promptly after recognition of severe disease, guided by likely pathogens and host risk factors. An early structured review at 24–48 h, integrating clinical evolution, microbiological results, and biomarkers, informs escalation, de-escalation, or discontinuation of therapy

**Fig. 2:**
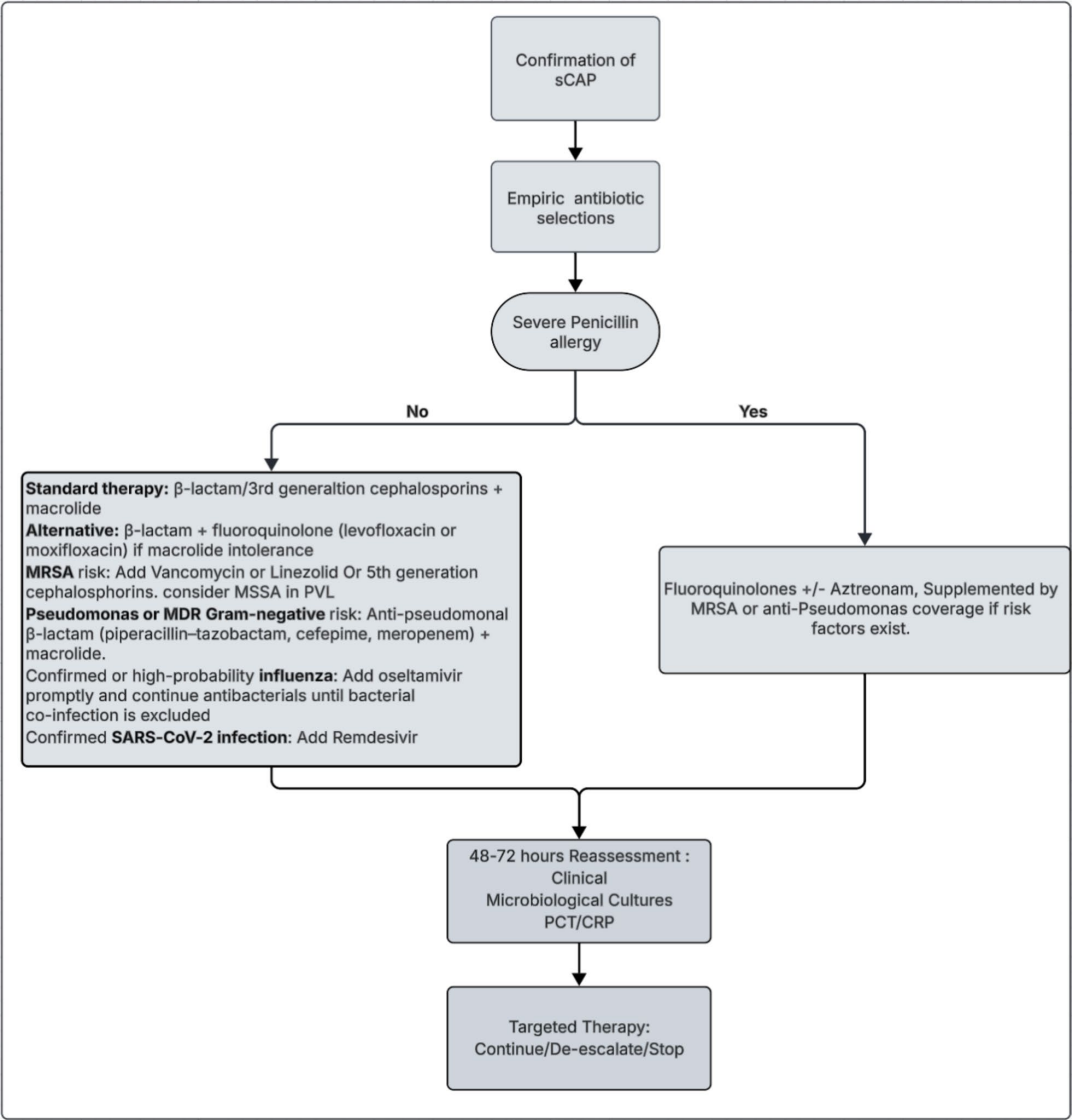
Framework for choosing empiric antibiotic therapy in scap. Empiric therapy for scap is guided by patient-specific factors, including β-lactam allergy and risk for MRSA or multidrug-resistant Gram-negative pathogens. Initial broad-spectrum therapy (β-lactam plus macrolide, or β-lactam plus respiratory fluoroquinolone if macrolide intolerance) may be escalated based on MRSA, Pseudomonas, or viral risk factors. In patients with severe β-lactam allergy, respiratory fluoroquinolone-based regimens with adjunctive coverage are recommended. Mandatory reassessment at 48–72 h, integrating clinical evolution, microbiology, and biomarkers (PCT/CRP), should guide continuation, de-escalation, or discontinuation of therapy. * penicillin allergy de-labelling [42]

In parallel with these guidelines, Martin-Loeches et al. propose a pragmatic, phenotype-driven framework (Figs. 1, 2) for empiric selection that integrates disease severity, comorbidities, and local resistance ecology [10].

Beyond established treatment regimens, several newer antimicrobials expand therapeutic options for challenging CAP phenotypes. Novel beta-lactam and beta-lactamase inhibitor combinations such as ceftazidime-avibactam and ceftolozane-tazobactam provide extended activity against resistant Enterobacterales and Pseudomonas species, supporting their use in selected high risk cases [43, 44]. Additional agents include newer macrolide solithromycin and the fluoroketolide cethromycin with improved potency against macrolide resistant Streptococcus pneumoniae [45].

Ceftaroline, an advanced cephalosporin with activity against MRSA, is licensed for CAP and has been evaluated as an option for severe presentations and cases involving MRSA when indicated [46]. Ceftobiprole represents another broad-spectrum cephalosporin with activity against MRSA and some activity against Pseudomonas, although its use remains dependent on national approvals and clinical context [47].

Lefamulin, a pleuromutilin antibiotic, demonstrated non inferiority to moxifloxacin in phase III trials for adult CAP with both intravenous and oral administration [48]. Omadacycline, an aminomethylcycline, also showed non inferiority to moxifloxacin in two pivotal trials [49]. It is important to emphasise that neither omadacycline nor lefamulin is currently validated for use in severe CAP, and their role remains limited to selected mild to moderate cases [48, 49].

These drugs may represent alternative options where intolerance, allergy, documented resistance, or specific safety concerns prevent the use of preferred first line agents. Antimicrobial stewardship remains essential. Their use should be restricted to clearly defined clinical indications rather than broad empirical application in all severe CAP cases [4, 10]. When multidrug resistant Gram-negative pathogens are suspected in severe CAP, therapy must be guided by microbiological documentation and local epidemiology. Empirical treatment with extended spectrum agents should be limited to patients with strong risk factors or severe septic presentations, followed by early reassessment and de-escalation once diagnostic information is available [10, 33].

Determining the optimal duration of antibiotic therapy in sCAP remains a critical component of antimicrobial stewardship. Fixed-duration regimens, traditionally 7–10 days for most pathogens, are supported by guideline recommendations and randomised trials showing that shorter courses (5–7 days) are non-inferior when patients demonstrate clinical stability [3, 4]. However, increasing attention has shifted toward biomarker-guided strategies, particularly using PCT or CRP, which enable earlier discontinuation in patients with favourable biomarker trajectories. Multiple trials and meta-analyses demonstrate that PCT-guided cessation safely reduces antibiotic exposure but require cautious interpretation given variability in biomarker kinetics and the higher recurrence signal, seen in a recent systematic review and meta-analysis [37]. To reconcile these approaches, several authors have proposed combination (“double-trigger”) algorithms, in which antibiotics can be discontinued once both clinical stability is achieved, and biomarker decline meets pre-specified thresholds (e.g., ≥ 80% reduction in PCT or absolute value < 0.25 ng/mL or reduction in CRP by 50%). This integrated strategy aligns with the current stewardship principles, balancing safety with the imperative to minimise unnecessary antibiotic use, particularly in the context of rising MDR pathogens.

## Co-adjuvant therapy

### Haemodynamic support

Severe community-acquired pneumonia (sCAP) represents not only a localised respiratory infection but a systemic inflammatory and circulatory disorder. Profound dysregulation of both humoral and cellular immune responses including marked cytokine release, endothelial injury, and the T-cell suppression, characteristics of severe sepsis drive the vasoplegia, capillary leak, and myocardial depression observed in septic shock. Early physiological studies by Natanson et al. demonstrated that the haemodynamic response to sepsis is characterised by high-output, low-systemic vascular resistance shock, with sepsis-induced myocardial depression contributing to impaired oxygen delivery despite preserved or elevated cardiac output [50]. These foundational insights underpin current principles of haemodynamic support in sCAP, emphasising early fluid resuscitation, vasopressor initiation, and perfusion-guided therapy.

According to the ATS/IDSA guidelines summarised by Metlay et al. episodes of sCAP complicated by shock or respiratory failure should be managed as sepsis syndromes in accordance with the Surviving Sepsis Campaign (SSC) principles [4]. Key components include:

- Early haemodynamic stabilisation with balanced crystalloids as first-line resuscitation fluids, avoiding excessive chloride load and reducing risk of renal dysfunction. The use of balanced crystalloids over normal saline, which has been associated with reduced kidney injury and improved outcomes in critically ill patients, including those with sepsis [51].
- Noradrenaline/Norepinephrine as first-line vasopressor, with vasopressin or adrenaline/epinephrine added if adequate mean arterial pressure (MAP ≥ 65 mmHg) cannot be maintained [52].
- Assessment for sepsis-induced cardiomyopathy, with bedside echocardiography to guide inotropic support, recognising that myocardial depression may occur even with normal preload [53].

These elements aim to maintain systemic perfusion, prevent multi-organ failure, and create physiologic conditions that support antibiotic delivery and gas exchange, forming a critical foundation for early survival in severe community-acquired pneumonia.

### Respiratory support

Respiratory failure is a defining feature of sCAP and often precedes ICU admission. Oxygenation strategies therefore play a central role in early management, with the goal of improving gas exchange, reducing work of breathing, and preventing progression to invasive mechanical ventilation (iMV). Over the last decade, high-flow nasal oxygen (HFNO) has transformed respiratory support in acute hypoxaemic respiratory failure by delivering heated, humidified gas at high flow rates with precise FiO₂ control, washout of anatomical dead space, and generation of low-level positive airway pressure. Compared with conventional oxygen, HFNO improves patient comfort, secretion clearance, and respiratory mechanics, allowing uninterrupted therapy during eating or conversation [54–57].

Randomised trials have demonstrated that HFNO may reduce intubation rates in selected patients with acute hypoxaemic respiratory failure, including pneumonia, when applied early with close monitoring [58]. However, treatment success is contingent on trajectory recognition. Persistent tachypnoea, rising work of breathing, or increasing FiO₂ requirement should prompt timely escalation to non-invasive or invasive support to avoid delayed intubation. Current guidance advises caution when using non-invasive ventilation (NIV) in de novo hypoxaemic failure, as inappropriate use has been associated with higher mortality likely due to patient–ventilator asynchrony and ventilator-induced lung injury, except in patients with COPD or hypercapnia where NIV remains first-line [59].

Awake prone positioning (APP) has emerged as a complementary non-invasive measure. By recruiting dorsal lung regions and improving ventilation-perfusion matching, APP enhances oxygenation and may delay or prevent intubation when well tolerated [60, 61]. Its benefit is greatest when used early and for prolonged sessions, particularly in combination with HFNO. However, evidence is still evolving and large RCTs are ongoing to determine which phenotypes benefit most. Together, HFNO and APP illustrate a modern respiratory strategy focused on physiological optimisation, patient tolerance, and prevention of ventilator-related complications.

When HFNO or non-invasive strategies fail, timely initiation of iMV is essential to prevent patient self-inflicted lung injury (P-SILI), progressive respiratory muscle exhaustion, and refractory hypoxaemia. Delayed intubation has been repeatedly associated with worse outcomes in severe pneumonia and ARDS, underscoring the need for close clinical monitoring and predefined escalation criteria [62, 63]. Once intubated, management follows lung-protective ventilation strategies similar to ARDS protocols with low tidal volume ventilation (6 mL/kg predicted body weight), plateau pressure < 30 cmH₂O, and adequate PEEP to prevent atelectrauma and improve oxygenation [64]. The application of PEEP should be individualised based on recruitability, hemodynamics, and driving pressure response. Prone positioning for ≥ 16 h/day is recommended in moderate–severe ARDS, which improves survival by enhancing dorsal lung recruitment and reducing ventilator-induced lung injury [64].

Neuromuscular blockade may be considered in early severe ARDS to improve ventilator synchrony and oxygenation, although routine prolonged use is discouraged due to risk of ICU-acquired weakness [65]. Conservative fluid management after initial resuscitation reduces ventilator days and risk of pulmonary oedema. Extracorporeal membrane oxygenation (ECMO) is reserved for refractory hypoxaemia despite optimal lung-protective ventilation and proning position, with evidence supporting its role as rescue therapy in carefully selected patients within experienced centres [66]. Together, HFNO, APP, NIV in selected phenotypes, and iMV with lung-protective strategies constitute a tiered respiratory support pathway in sCAP. The objective is not solely to oxygenate but to minimise ventilator-induced injury, avoid delayed intubation, and individualise support according to evolving respiratory mechanics and host response.

### Corticosteroids

Corticosteroids have long been considered an adjunctive strategy in severe community–acquired pneumonia, aiming to suppress excessive inflammation, preserve endothelial integrity, and enhance cardiovascular stability [67–69]. In addition to their genomic anti-inflammatory actions, corticosteroids exert rapid non-genomic effects, including improved expression and function of adrenergic receptors, which can augment vasopressor responsiveness in shock states [68, 70]. Early evidence was inconsistent, largely due to heterogeneity in patient selection, dosing strategies, timing, and clinical endpoints [71, 72]. More recent high-quality randomised trials have helped clarify their role, although uncertainty remains regarding which populations derive the greatest benefit [73–75].

Martin-Loeches et al. synthesised contemporary evidence showing that adjunctive corticosteroid therapy in sCAP reduces time to clinical stability, improves oxygenation, and may prevent progression to septic shock or acute respiratory distress syndrome, with the greatest effect observed in critically ill patients with marked systemic inflammation and hypoxaemia admitted to the ICU [76]. A broader review by Martin-Loeches and Torres which also examined influenza pneumonia and COVID-19 highlighted the importance of precise patient selection. While corticosteroids appear beneficial in severe bacterial CAP and clearly reduce mortality in hypoxaemic COVID-19, observational studies consistently suggest potential harm in influenza, including increased mortality and secondary infections [77].

The CAPE COD multicentre RCT provided important evidence by demonstrating that adjunctive hydrocortisone 200 mg per day for 4 to 8 days significantly reduced 28-day mortality and treatment failure in severe community acquired pneumonia with shock requiring oxygen therapy or mechanical ventilation, without increasing secondary infections or gastrointestinal bleeding. These findings established hydrocortisone as the most evidence supported corticosteroid in this setting [73]. Reyes et al. commented that although corticosteroids represent progress, uncertainty remains regarding optimal duration, phenotype targeting, and integration with emerging immunomodulatory therapies [78].

A growing understanding of immune pathobiology provides additional context. The immune system attempts to restore homeostasis by balancing disease resistance, which eliminates pathogens but may cause tissue damage, and disease tolerance, which limits host injury without reducing pathogen burden [79]. Corticosteroids act broadly across these pathways and although they may reduce shock related cardiovascular dysfunction as much as inflammatory injury, their non-specific effects include risks such as hyperglycaemia, secondary infections, gastrointestinal bleeding, and potential increased readmission rates. Many meta-analyses are based on older trials, with varied definitions, early termination, and limited power. Importantly, most trials exclude immunosuppressed patients (e.g., transplant recipients, cancer therapy, chronic steroids), limiting the generalisability of results to these high-risk groups and there remains minimal evidence to support corticosteroid use in non-severe community acquired pneumonia [80, 81].

The REMAP CAP platform trial found that a 7 day course of hydrocortisone did not reduce mortality in patients with severe community acquired pneumonia although smaller benefits and possible harm are not excluded [75]. However, when incorporated into updated meta-analyses, the conclusion that corticosteroids reduce short term mortality was maintained but not long-term [82]. Interpretations are complicated by overlap between severe community acquired pneumonia, septic shock, and acute respiratory distress syndrome, where corticosteroids may be used for reasons distinct from pneumonia itself. The SONIA RCT also assessed hydrocortisone in severe CAP but was limited by substantial heterogeneity, imprecise phenotype classification, and methodological concerns. Although widely discussed, the trial’s multiple biases and analytic limitations mean its findings should be interpreted cautiously [74].

Current guidelines reflect this nuance. The ERS/ESICM/ESCMI/ALAT 2023 guideline recommends corticosteroids as adjunctive therapy in severe bacterial community acquired pneumonia requiring ICU admission or respiratory support, citing moderate quality evidence for mortality reduction and faster recovery [5]. Recent ATS guidance advises against systemic corticosteroids in non-severe community acquired pneumonia and suggests use in severe bacterial pneumonia except in influenza, where evidence indicates potential harm and randomised controlled trials are lacking [41]. Both US and European guidelines remain cautious in non-shock inflammatory phenotypes and emphasise precision based rather than universal application [4, 5].

In summary, corticosteroid therapy in severe community acquired pneumonia has progressed from controversy toward a more refined consensus. Corticosteroids are now supported in selected severe bacterial cases with shock or high systemic inflammation admitted to ICU. Future studies should focus on patient phenotypes, prognostic enrichment, and identification of treatable traits rather than syndromic labels, to define who benefits and to avoid growing risks of indiscriminate steroid overuse.

### Immunomodulation and personalised approaches

The recognition that sCAP involves heterogeneous immune dysregulation rather than a uniform hyperinflammatory state has led to the exploration of personalised immunotherapies that may benefit selected patients. Individuals may present with divergent immune responses, ranging from cytokine-driven hyperinflammation to profound immunosuppression or “immunoparalysis.” Recent work has proposed structured bedside approaches to assess immune function directly, supporting the concept that treatment should be aligned with the patient’s immune phenotype rather than applied uniformly across all presentations [83]. This variability has prompted the development of personalised immunomodulatory strategies aimed at tailoring therapy to host immune trajectories rather than relying on syndromic labels. Supporting this concept, Martin-Loeches et al. demonstrated that the protective association of endogenous immunoglobulin levels against sepsis mortality is restricted to patients with moderate organ failure, suggesting that only specific immunological phenotypes stand to benefit from immunoglobulin-based interventions and that indiscriminate administration may not be advantageous [84]. Such findings underscore the need to identify patients whose humoral immunity is impaired yet still responsive to adjunctive support.

One of the most studied immunomodulators in this context is trimodulin, a polyvalent immunoglobulin preparation containing IgM, IgA, and IgG. In a multicentre, randomised, double-blind, placebo-controlled phase II trial, Welte et al. demonstrated that adjunctive trimodulin therapy in patients with sCAP and low baseline IgM levels was associated with lower mortality, reduced duration of mechanical ventilation, and shorter ICU stays compared with placebo. The proposed mechanisms include enhanced opsonisation and complement activation, restoration of humoral immunity, while suppressing dysregulated cytokine release [85]. Despite these promising results, uptake of immunoglobulin-based therapies such as trimodulin remains limited in many healthcare systems, including Japan, where immunoglobulin preparations are infrequently used in pneumonia. As further phase III trials and real-world data emerge, practice patterns may evolve, but at present these agents should still be regarded as emerging, phenotype-targeted adjuncts rather than standard of care.

More recently, the research has shifted toward immune phenotyping to identify subgroups of pneumonia patients who may benefit from targeted immunotherapies. Martin-Loeches et al. highlighted distinct immunological phenotypes such as hyperinflammatory, hypoinflammatory, and mixed, characterised by variations in cytokine patterns, lymphocyte exhaustion markers, and innate immune responsiveness. Such immune profiling has revealed that some sCAP patients exhibit sustained immunosuppression with reduced monocyte HLA-DR expression, impaired neutrophil function, and lymphopenia features associated with secondary infection and late mortality [86].

Taken together, these findings reflect a broader shift in severe pneumonia care from a pathogen-centred to a host-response-centred paradigm, where supporting cardiovascular stability and modulating inflammation are as vital as eradicating infection.

### Future directions

The management of sCAP is evolving toward precision medicine where treatment is tailored not only to the pathogen but also to the host immune profile and dynamic risk trajectory. Current approaches still depend heavily on syndromic definitions, conventional microbiology, and a limited panel of biomarkers. However, several emerging tools promise to transform this landscape.

First, advances in integrated pathogen and host response diagnostics could substantially reduce the proportion of microbiologically undiagnosed sCAP. Unbiased metagenomic next generation sequencing and multiplex molecular platforms are being evaluated in lower respiratory samples to detect fastidious, atypical, and mixed infections while simultaneously characterising host transcriptomic responses. These approaches may enable discrimination between infectious and non-infectious causes of respiratory failure and between viral and bacterial triggers [87]. This could support earlier de-escalation of broad-spectrum antibiotics, more targeted use of antivirals and antifungals, and more appropriate selection of patients for adjunctive immunomodulatory therapy.

Second, host immunophenotyping and endotyping are likely to play a key role in guiding personalised therapies. Increasing recognition of biological heterogeneity from hyperinflammatory states to immune paralysis supports the concept that adjunctive treatments such as corticosteroids, trimodulin, immunoglobulins, or cytokine directed therapies should be deployed based on specific immune signatures rather than uniformly. Host response classifiers derived from blood transcriptomic or proteomic signatures are under development to stratify patients according to expected benefit or risk. This mirrors advances in sepsis and acute respiratory distress syndrome where biologically defined subphenotypes have demonstrated differential responses to therapies in secondary analyses of randomised trials [84–86, 88].

Third, adaptive and platform trial designs offer an efficient infrastructure to evaluate multiple interventions across diverse severe community-acquired pneumonia phenotypes. Randomised embedded multifactorial adaptive platform trials such as REMAP-CAP demonstrate how continuously learning systems can test different antimicrobial regimens, immunomodulators, and ventilatory strategies, updating allocation probabilities in near real time as evidence accumulates. [89–91]. However, their strengths in efficiency and pragmatic generalisability do not always translate into clear mechanistic insights. The recent SONIA RCT illustrates how pragmatic designs with broad inclusion criteria and limited biological characterisation may generate more uncertainty than clarity when evaluating immunomodulatory therapies [74]. If the aim is to identify well-defined biological subphenotypes and determine which immune-targeted or corticosteroid strategies benefit specific groups, high-resolution explanatory RCTs with rigorous immune and clinical phenotyping are indispensable. Embedding both approaches, pragmatic platforms for real-world evaluation and explanatory trials for mechanistic precision into ICU research pathways will be critical to resolving questions on optimal corticosteroid strategies, combinations of immune-directed therapies, and biomarker-guided antibiotic durations within clearly delineated biological groups.

Finally, implementation science and global equity are essential to ensure that these advances translate into improved survival. Although high-income regions move toward metagenomics, transcriptomics, and AI-based risk stratification [92, 93], many centres in low- and middle-income settings lack access even to basic microbiology, imaging, or ICU beds, underscoring the critical role of implementation frameworks in bridging this gap [15, 94]. Future work must therefore combine innovation with scalable severity scores, biomarker-based triage tools, and antimicrobial stewardship bundles that can be adapted to resource constrained environments. Core principles remain constant: early recognition, rapid but reviewable empiric treatment, thoughtful adjunctive therapy, and systematic reassessment.

Together, these developments highlight a transition from a uniform management strategy to a biologically informed adaptively tested and context aware model of care. The goal is clear: the right patient receiving the right therapy at the right time and for the right duration.

## Conclusion

Severe community acquired pneumonia is a complex systemic syndrome in which outcomes depend on both pathogen factors and the host immune response. Despite advances in antimicrobials, diagnostics, and supportive care, sCAP remains a major cause of ICU admission and mortality. Early recognition and rapid, appropriate treatment are essential, as progression to shock and multiorgan failure strongly influences prognosis. Accurate diagnosis continues to be challenging due to mixed and unidentified pathogens. Structured respiratory sampling and multiplex PCR significantly improve pathogen detection, while biomarkers such as procalcitonin and CRP support treatment monitoring and safe antimicrobial stewardships when used within clinical context. Severity assessment benefits from combining clinical scoring tools, radiologic severity, and biomarker dynamics to inform triage and treatment intensity.

Optimal management follows a dynamic pathway to include immediate empiric therapy based on severity and risk factors, followed by formal reassessment at 24 to 48 h guided by microbiology and clinical evolution. Adjunctive strategies, including evidence-supported corticosteroids in severe bacterial CAP with shock requiring respiratory support, reflect growing recognition that modifying host response is as important as pathogen control. However, corticosteroids should not be used in influenza-associated pneumonia or in immunosuppressed patients, where available evidence suggests potential harm or lack of benefit.

Looking ahead, precision and phenotype-based care represent the future. Integration of rapid pathogen diagnostics, immune profiling, biomarker-guided duration of antibiotics, and adaptive clinical trial platforms will be key to resolving current uncertainties and avoiding unnecessary antimicrobial use. A coordinated multidisciplinary model that unites early recognition, diagnostic precision, targeted therapy, and systematic reassessment offers the best opportunity to improve survival and deliver personalised care in severe community acquired pneumonia.

## Data Availability

Not applicable. No original data were generated or analysed for this review.

## Abbreviations

ARDS: Acute respiratory distress syndrome
BUN: Blood urea nitrogen
CAP: Community-acquired pneumonia
CRP: C-reactive Protein
DIC: Disseminated intravascular coagulation
HFNO: High-flow nasal oxygen
ICU: Intensive care unit
IMV: Invasive mechanical ventilation
NIV: Non-invasive ventilation
PaO₂/FiO₂: Ratio of arterial oxygen partial pressure to fraction of inspired oxygen
PCR: Polymerase chain reaction
PCT: Procalcitonin
RSV: Respiratory syncytial virus
sCAP: Severe community-acquired pneumonia
WBC: White blood cell count
